# Extensions of Granger Causality Calculations on Brain Networks for Efficient and Accurate Seizure Focus Identification via iEEGs

**DOI:** 10.3390/brainsci11091167

**Published:** 2021-09-01

**Authors:** Victor B. Yang, Joseph R. Madsen

**Affiliations:** 1 School of Medicine, The Johns Hopkins University, Baltimore, MD 21205, USA; 2 Division of Epilepsy Surgery, Department of Neurosurgery, Boston Children’s Hospital, Harvard Medical School, Boston, MA 02115, USA

**Keywords:** network centrality, epilepsy surgery, seizure networks, intracranial EEG, surgical planning, Monte Carlo sampling

## Abstract

Current epilepsy surgery planning protocol determines the seizure onset zone (SOZ) through resource-intensive, invasive monitoring of ictal events. Recently, we have reported that Granger Causality (GC) maps produced from analysis of interictal iEEG recordings have potential in revealing SOZ. In this study, we investigate GC maps’ network connectivity patterns to determine possible clinical correlation with patients’ SOZ and resection zone (RZ). While building understanding of interictal network topography and its relationship to the RZ/SOZ, we identify algorithmic tools with potential applications in epilepsy surgery planning. These graph algorithms are retrospectively tested on data from 25 patients and compared to the neurologist-determined SOZ and surgical RZ, viewed as sources of truth. Centrality algorithms yielded statistically significant RZ rank order sums for 16 of 24 patients with RZ data, representing an improvement from prior algorithms. While SOZ results remained largely the same, this study validates the applicability of graph algorithms to RZ/SOZ detection, opening the door to further exploration of iEEG datasets. Furthermore, this study offers previously inaccessible insights into the relationship between interictal brain connectivity patterns and epileptic brain networks, utilizing the overall topology of the graphs as well as data on edge weights and quantity of edges contained in GC maps.

## 1. Introduction

Epilepsy is a chronic neurological disorder whose only known cure is surgery to resect the brain’s seizure onset zone (SOZ, which is often not entirely or exactly removed; the part of the brain actually removed during surgery is known as the resection zone—RZ). However, such surgery requires determination of the RZ and SOZ via a lengthy, inconvenient period of invasive iEEG (intracranial EEG) monitoring. This is because neurologists and neurosurgeons must wait for an ictal event (e.g., a seizure) in order to accurately identify the iEEG electrodes that report abnormal activity and plan for resection surgery. Armed with iEEG recordings of multiple ictal events, physicians inspect these data by eye, which is itself an arduous process. Instead, we look to use interictal iEEG recordings as a basis for surgical planning. If interictal (i.e., everyday activity not from a seizure) iEEG data alone could be used to determine the SOZ, then the SOZ determination phase could be theoretically reduced from at least a week to a matter of hours, which is the time it takes to implant electrodes, run the algorithm, and take out electrodes. It is important to note that this reduction in resection planning time is theoretical and dependent on maintaining a rigorous planning process. In any case, since the current standard of care involves inspection of EEG data streams by the naked eye, this project aims to develop an algorithm that standardizes this process from case to case, further saving physician time and boosting accuracy of SOZ prediction.

The problem of the algorithmic seizure onset zone detection using only interictal data has been approached from a wide variety of angles, apart from GC. Perhaps the most developed is the use of interictal high-frequency oscillations (80–500 Hz; HFOs) derived from iEEG and scalp EEG recordings. HFOs have demonstrated potential in their ability to serve as a biomarker for the seizure onset zone [1,2,3,4]. Localized HFOs have also been detected from ictal sEEG recordings, supporting the case for the accuracy of HFOs in SOZ detection [5]. However, despite recent advances in algorithmic HFO detection, the standard protocol remains physician determination of HFO through visual inspection of EEG data [3,6]. Furthermore, the HFO methods require processing data in the frequency domain, which is more computationally intensive than the more recent GC time-domain method [1]. Because of our motivations to cut down on the time needed for SOZ determination, eliminating computational steps and automating the entire process are important for the clinically viability of any SOZ determination methods. Thus, this study builds upon the GC approach, which require neither physician inspection of EEG data nor time-intensive frequency domain processing.

Others have applied variants of the Granger Causality algorithm or graph algorithms that we study to seizure onset detection. Whereas we look at pairs of nodes in our analysis, studies have looked at triplets to determine whether inclusion of a third node enhances the information shared between two other nodes in the same network, called synergistic effects [7]. These have had some success, finding that synergistic effects in the immediate pre-ictal period correspond to nodes in the SOZ [8]. Combining this triplet approach with our pairwise one is a potential future area of study. Still others have applied frequency-domain GC in analysis of pre-ictal data and ranked eigenvector centrality in analysis of ictal data to determine the SOZ [9,10]. However, none of these studies investigated the relationship between baseline interictal data and ictal data to predict the SOZ.

Still others have adopted network modeling techniques for interictal prediction of the SOZ. These studies have also utilized EEG recordings of interictal patient brain activity [11,12]. Demonstrating the difficulty of developing SOZ identification tools that generalize across a larger cohort of patients, one such study found success in identifying robust chimera state markers in interictal recordings for one patient out of a cohort of 15 patients [11]. We present similarly generalizable SOZ identification algorithms for all epilepsy cases with a much higher success rates. However, due to the diverse nature of epilepsy onset in different regions of the brain and from differing etiologies, identifying a specific subset of clinical cases for which a SOZ identification tool is most useful will likely improve the future usefulness of the tools analyzed both in this study and others. Another study, also based on interictal EEG recordings, interestingly develops a predictive tool to simulate results of resection of any node in a brain’s network [12]. Their study differs from ours in that a significant portion of patients (7 of 16) did not have successful surgical outcomes [12]. Without a definitive source of truth (i.e., neurologist-identified SOZ via the current standard of care), assessing the accuracy of a novel SOZ identification tool becomes necessarily speculative. Thus, another contribution of our study is a robust cohort of 24 patients who underwent resection surgery. All 24 had at least a worthwhile improvement (Engel class III or above), and 22 were free of disabling seizures (Engel class I). Nevertheless, in the cited study, even amongst the nine cases with successful surgical outcomes, the predicted SOZ inconsistently matched the actual SOZ [12], mirroring the difficulties we encountered in our own study. Larger patient cohorts created through a combination of data across studies could prove fruitful for homing in on subsets of patients for which each SOZ/RZ prediction tool offers accurate predictions. We have included data on demographic information, clinical background, and seizure localization for the cohort of patients in this study in Tables S1 and S2.

Specifically, we study electrocorticography (ECoG) recordings, a form of intracranial EEG (iEEG) monitoring where a skull flap is removed and a grid of electrodes is placed directly onto the brain’s surface. Our population of 25 patients had an average of 102.56 grid electrodes (often referred to as “nodes”) implanted for invasive monitoring. The neurologist-determined SOZ contained an average of 19.62 nodes and the RZ contained an average of 21.88 nodes. One of the 25 patients did not undergo resection surgery after invasive monitoring, resulting in 24 patients with an actual RZ. Investigations of other iEEG recording types (including stereotactic EEG, or sEEG) as they pertain to epilepsy surgery will be the likely subject of later studies by our group.

We build off the previous result that time domain Granger Causality (GC) analysis of baseline interictal iEEG data indicates which electrodes seem to be more influential for activity at a network of electrodes [1]. The retrospective study found that brain regions surrounding these interictally causal electrodes matched the regions chosen for resection through traditional analysis of ictal data with an aggregate probability much smaller than chance (*p* < 10^−20^) [1]. GC analyses assign a metric for every pair of electrodes to measure the degree to which activity at one electrode dictates activity at another. This creates a graph of the patient’s brain, also known as a GC map. From the GC map, summation of all the GC values originating at each electrode results in computation of a total GC outdegree metric for that electrode. Nodes are then ranked by their total GC outdegree and the ranks of nodes belonging to the actual SOZ and RZ are summed to create a rank order sum metric. Via comparison of this sum to a random distribution of rank order sums of the same size subset from the same starting number of ranks, it is shown that total GC outdegree results in a statistically significant rank order sum of actual SOZ and RZ nodes with an aggregate probability across all 25 patients much lower than predicted by chance [1]. However, because the total GC outdegree metric was previously applied without evaluation of alternatives, we start with the GC map and aim to create a more precise algorithm that informs selection of a RZ/SOZ. For ease of comparison, we also use the rank order sum method to evaluate algorithms’ predictive capability. It is important to point out that the Granger statistical approach demonstrates that a particular signal in one channel statistically follows a signal in a different channel. This does not in fact prove that one event is causal, but the term ‘Granger Causality’ is widely used as the name of the statistic. We will keep the name for clarity with this caveat, understanding that the current study begins with the connectivity data derived and reported earlier [1].

This study comprises three angles from which the above problem is tackled. These were motivated by numerous reasons, chief among them a desire to explore methods that built upon previous GC techniques without adding significant algorithm runtime as well as attempts to naturally mimic brain signals traveling through the network of the GC map. First is a Monte Carlo sampling approach that visits nodes with probabilities weighted by the GC map. Second is an application of Google’s PageRank algorithm where nodes take the place of webpages and are assigned a metric of importance relative to other nodes. Third are centrality algorithms that aim to tease out the subset of nodes most important for transmission of information through the brain’s network. Variants of each algorithm are developed, fine-tuning their application to the problem of RZ/SOZ determination and to the goal of revealing connectivity patterns in an epileptic patient’s brain.

## 2. Materials and Methods

### 2.1. Patients

For comparison purposes, we utilized the same 20 min interictal iEEG recordings of a population of 25 patients exhibiting medically intractable epilepsy used by Park and Madsen, 2018. After careful review by the multidisciplinary Epilepsy Surgery Conference, each patient underwent long-term monitoring in preparation for resection surgery with the epilepsy team at Boston Children’s Hospital. The goal of monitoring was to obtain iEEG data necessary for accurate SOZ determination. Ultimately, resection surgery was performed in 24 out of 25 patients in this cohort on the basis of data obtained through both iEEG recordings and less invasive means. Because of the retrospective nature of our study, we obtained Institutional Review Board approval without requiring patient consent.

### 2.2. Granger Causality Map Computation

GC maps were computed for each patient using MATLAB’s Granger causal connectivity analysis (GCCA) toolbox [13]. Due to the computationally intensive nature of each run of a Granger Causality algorithm, 20 s segments of iEEG data were processed at a time. There were 60 separate time segments analyzed and 60 GC maps created for each patient for a total of 20 min of data analyzed. The 20 s segments were selected randomly from interictal portions of a week of invasive monitoring. For each 20 s segment, each computed GC map is then viewed as a network (i.e., graph, which is hereafter referred as G) with each iEEG electrode recording point representing a node and edge weights determined by the values contained in the GC map. Next, an F Test is applied to the GC matrices in order to identify and eliminate edges that do not achieve statistical significance [1].

### 2.3. Graph Algorithms

#### 2.3.1. Monte Carlo Sampling Approach

From a broad perspective, this algorithm visits nodes one by one, with each step determined by probabilities defined by GC map edge weights. Nodes’ importance to the epileptic network is thus decided according to their number of visitations.

A Monte Carlo Sampling metric is computed for each patient using a Python script implementing the algorithm as follows. A node n∈V is selected at random from a graph $G=\left(V,E\right)$ and becomes the current node $n_{1}$. A new current node $n_{2}$ is then selected with probabilities $\left\{p_{1},\;p_{2},\;\ldots ,\;p_{\left|V\right|}\right\}$, the set of GC matrix edge weights originating from $n_{1}$. Note that these probabilities are normalized such that all the edge weights add up to one. Then, another current node $n_{3}$ is recursively selected using probabilities derived by the weights of the GC matrix edges leaving $n_{2}$. This process continues until 1000 nodes have been visited. Then, another node n∈V is selected at random from G and the process continues all over again. For each GC matrix, this process happens 1000 times, each time with a random selection of one node followed by up to 999 subsequent visitations according to GC matrix probabilities. Across all of the up to 1000 × 1000 visitations of individual nodes, a dictionary keeps track of how many times each node is visited. It is hypothesized that nodes with a greater number of visitations are more central to the network and thus should be prioritized for inclusion in the RZ/SOZ.

It is important to note that although 1000 represents the maximum number of iterations possible, some nodes do not contain any outdegree edges. Visitation of such a node prematurely stops the chain of visitations and moves the algorithm onto the next random restart. The F Test applied to GC matrices results in elimination of edges that do not achieve statistical significance; thus, many nodes do not contain outward edges [1].

Five separate variants of this approach are independently tested.

The number of visitations per random restart, originally 1000, is tested at various orders of magnitude: 100, 1000, 10,000.

The number of random restarts, originally 1000, is also tested at various orders of magnitude: 100, 1000, 10,000.

Edges in the GC map are reversed, such that an edge weight describing how much activity at a node $n_{i}$ causes activity at a node $n_{j}$ now describes how much activity at $n_{j}$ causes activity at $n_{i}$. It is hypothesized that when edges are reversed, sampling tokens now travel “uphill” towards the node that instigated neural activity as opposed to the node that exhibits the “downhill” activity itself. In this variant, “uphill” nodes would receive a greater number of visitations and a higher ranking.

Instead of keeping track of total visitations, an algorithm variant instead keeps track of the average interval between visitations for each node. For example, if node $n_{1}$ is visited, then three other nodes are visited before $n_{1}$ is next visited again; 4 would be recorded as a visitation interval for $n_{1}$. Intervals are averaged for each node and nodes are ranked from shortest average interval to longest. It is important to note that visitation intervals do not carry over in the event of a random restart (i.e., a random restart resets visitation intervals for all nodes to zero and does not impact nodes’ final rankings). This limits the possibility that nodes achieve a higher ranking through visitations purely due to chance.

Multiple nodes are sampled at the same time, such that one can imagine more than one token simultaneously traversing a graph. In this algorithmic variant, a visitation is only recorded if all tokens are visiting the same node at the same time.

#### 2.3.2. PageRank Approach

PageRank once was the website ranking system used by Google’s search engine, determining the importance of each website in order to display relevant results [14]. An analogous problem is the ranking of iEEG electrode contacts by their importance to a patient’s epileptic network. Computed using the Python network library implementation, the algorithm is explained here through a simplified example. Let P represent the entire internet. In our internet, there are three webpages: $p_{1}$, $p_{2}$, and $p_{3}$. There are also only three total links across all three webpages: $p_{1}$ links to $p_{2}$ and $p_{3}$, and $p_{2}$ links to $p_{1}$. Each webpage starts off with an equal PageRank metric, which we will call 10. In each iteration of the algorithm, each page evenly divides all of its PageRank metric and transfers it to all of the links it is connected to. This can be expressed mathematically as $PageRank\left(p_{i}\right)=\underset{p_{j}\in A_{p_{i}}}{\sum }\frac{PageRank\left(p_{j}\right)}{Out\left(p_{j}\right)}$, where $A_{p_{i}}$ represents the set of all nodes with an edge heading into $p_{i}$ and $Out\left(p_{j}\right)$ represents the outdegree of node $p_{j}$. Note that this is the computation for a single node $p_{i}$ for a single iteration of PageRank. In the case of a node with no outward edges, the node’s PageRank is evenly distributed amongst all other nodes. So, in our example, $p_{1}$ transfers 10/2 = 5 to $p_{2}$ and 5 to $p_{3}$, and at the same time $p_{2}$ transfers 10/1 = 10 to $p_{1}$. Because $p_{3}$ has no outward edges, 5 is transferred both to $p_{1}$ and to $p_{2}$. At the end of the first iteration of the algorithm, we have 15 for $p_{1}$, 10 for $p_{2}$, and 5 for $p_{3}$. In the second iteration of the algorithm, $p_{1}$ transfers 15/2 = 7.5 to $p_{2}$ and 7.5 to $p_{3}$, $p_{2}$ transfers 10/1 = 10 to $p_{1}$, and $p_{3}$ transfers 2.5 to $p_{1}$ and to $p_{2}$. At the end of the second iteration of the algorithm, we have 12.5 for $p_{1}$, 10 for $p_{2}$, and 7.5 for $p_{3}$. This is continued until the algorithm converges, at which time pages are ranked by their PageRank metric from highest to lowest.

There are a couple of small caveats. First is the damping factor, δ < 1, that is set to 0.85 by default. For each iteration, every node’s PageRank metric is multiplied by the damping factor to encourage convergence. Second is that instead of a node evenly dividing its PageRank metric amongst all the nodes it connects to, it divides its PageRank metric according to a ratio specified by its outward edge weights. This is useful for the application of PageRank to SOZ identification because the weights of PageRank are now set according to the GC map. Finally, similar to the Monte Carlo Sampling approach’s motivations and implementation, a PageRank variant with reversed edges is attempted.

#### 2.3.3. Centrality Approach

Centrality algorithms were developed to analyze social networks for identification of their most influential members [15]. In this study, all centrality algorithms are computed using the Python network library implementation.

Betweenness centrality is computed as follows. First, the algorithm finds all shortest paths between all pairs of nodes in the graph. Note that this takes edge weights into account in an inverse manner, where a larger edge weight means a “shorter”, or easier-to-take, path. For each node, the betweenness centrality metric becomes the fraction of shortest paths that pass through that node. The idea motivating this algorithm is that signals, cars, people, etc. are all more likely to take the shortest path over a longer one to get somewhere, and that the shorter the paths a node is involved in, the more central that node can be said to be. Betweenness centrality can be expressed mathematically as $betweenness\left(n\right)=\underset{\forall p,q\neq n}{\sum }\frac{\sigma_{pq}\left(n\right)}{\sigma_{pq}}$, where $\sigma_{pq}$ represents all the shortest paths from p to q (taking into account ties for shortest path) and $\sigma_{pq}\left(n\right)$ represents all the shortest paths from p to q that pass through n [15].

The harmonic centrality metric for a node n is calculated by taking the summation of inverse distances between n and every other node in the network, normalized to the total number of nodes in the network [16]. This can be expressed mathematically as $harmonic\left(n\right)=\frac{\sum_{\forall p\neq n}\frac{1}{L_{pn}}}{N-1}$, where $L_{pn}$ represents the length of the shortest path from node p to node n and N represents the total number of nodes in the network [16].

The final two centrality algorithms tested, indegree and outdegree, are motivated by the observation that a high total GC outdegree at a node n can be due to two factors: first, the outward edges originating from n can have larger weights and, second, n can simply have more outward edges. Because an F test of statistical significance is applied to GC matrices before computation of additional algorithms [1], thereby zeroing out any edges that seem to be the product of noise, the second factor is hypothesized to be a potential difference-maker for identification of causal SOZ, RZ, and SOZ ∩ RZ nodes versus unremarkable nodes. If a node has a greater number of outward or inward edges, measured by outdegree centrality and indegree centrality, then it is hypothesized to be more likely to instigate epileptic activity at other nodes.

### 2.4. Statistical Analysis

**Figure 1:**
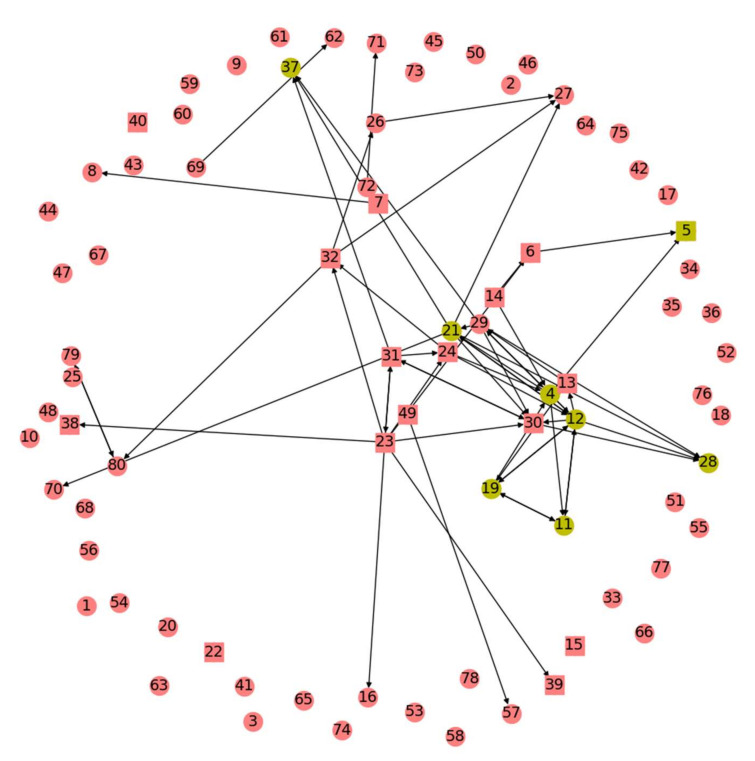
Results of one run of any of the graph algorithms tested can be visually depicted as shown. In this example, displayed are the Indegree Centrality results for one patient for one run of the algorithm. Axes here are arbitrary units from the GC map. Each number labels a node, with a square representing those in the SOZ and a circle representing all others. The top eight nodes by Indegree Centrality metric are yellow. Directed edges are shown. Figure produced using matplotlib.

Each of the graph algorithms tested returns a metric for each node’s importance to the epileptic network. Nodes are then ranked according to this metric, as shown in Figure 1, where all the ranked nodes for a single patient are displayed along with the largest magnitude edge weights from the GC map. The graph algorithm used to create such a ranking is evaluated via the rank-order sum method as follows [1]. First, each node is assigned a metric (i.e., number) by the algorithm under evaluation. For example, the Monte Carlo sampling metric is the number of visitations each node receives. The nodes are then ranked sequentially according to their metric, such that $\forall n\in V,\;1\leq r_{n}\leq N$, where the patient in question is being recorded by N nodes and $r_{n}$, is the rank of a node n. A rank of 1 corresponds to the node with the highest metric of all nodes and a rank of N with the lowest. The rank order sum of neurologist-determined RZ and SOZ nodes (this acts as the true RZ and SOZ against which algorithmically determined RZs and SOZs are validated) is computed by adding the ranks of all the nodes in the RZ and SOZ, respectively. This rank order sum is compared to a normal distribution of possible rank order sums created via Monte Carlo simulation for a subset of size $\left|SOZ\right|$ from N total nodes. In other words, the normal distribution of rank order sums is obtained by selecting $\left|SOZ\right|$ integers from the set $\left\{1,2,3,\ldots ,N-1,N\right\}$ and summing them. This process of selecting and summing integers is repeated 10^5^ times, thus creating the normal distribution [17]. In order to determine significance of the experimental rank order sum from the algorithm being tested, if the experimental sum is significantly less than the sum as expected by chance from the normal distribution of sums, then it can be concluded that the algorithm reveals information about the causality of each node and the makeup of the RZ and SOZ [1].

**Figure 2:**
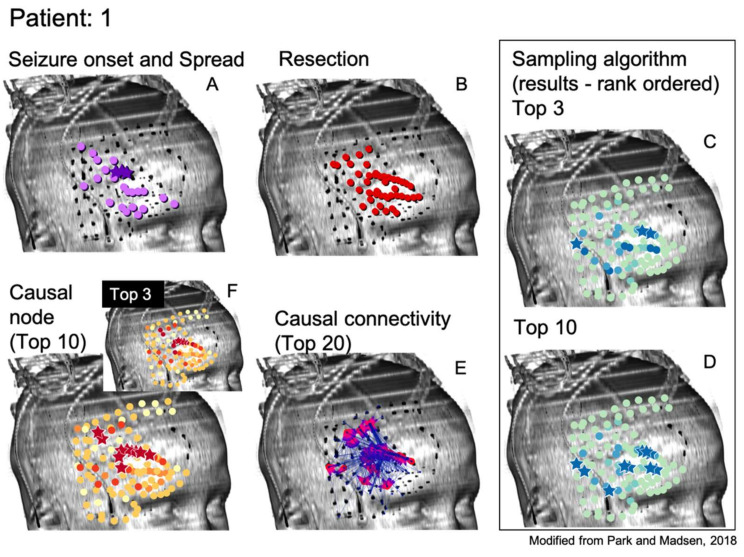
Results for the Monte Carlo sampling algorithm (1000 random restarts, 1000 samples per random restart) run on one patient are shown. Shown are the neurologist-determined SOZ (panel **A**) and surgical RZ (panel **B**) determined through means currently utilized for epilepsy surgery. The top 3 (panel **C**) and top 10 (panel **D**) ranked nodes according to the sampling algorithm are displayed. Note the similarities between top sampling nodes and neurologist-determined SOZ and RZ nodes. (Panel **E**) shows the most influential edges of the GC map for reference. It is along such edges that tokens travel in the sampling algorithm. (Panel **F**) shows the top 3 and 10 nodes as determined by the total GC outdegree method, also for reference [1].

**Figure 3:**
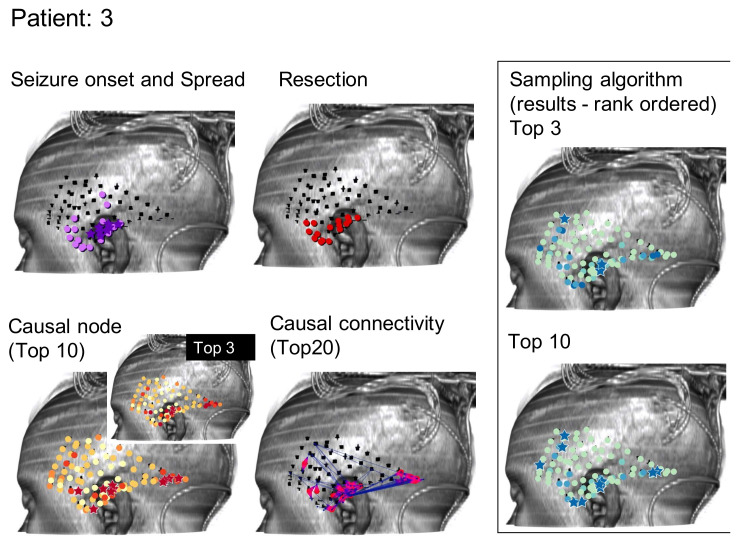
Similar to Figure 2, results for the Monte Carlo sampling algorithm (1000 random restarts, 1000 samples per random restart) run on one patient are shown.

**Figure 4:**
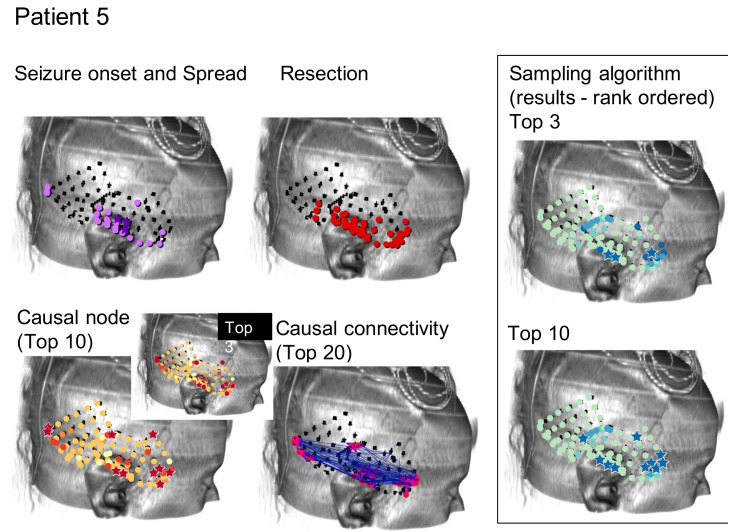
Similar to Figure 2 and Figure 3, results for the Monte Carlo sampling algorithm (1000 random restarts, 1000 samples per random restart) run on one patient are shown.

As can be seen, the algorithms being tested in this manner do not immediately suggest a resection zone; rather, they create a ranking of nodes where the nodes in the SOZ are much more likely to be ranked first. Methods can be devised to select a SOZ from this ranking, such as selecting the top 3 or 10 ranked nodes as shown from one patient by the starred nodes in Figure 2C,D, respectively. This is compared to the neurologist-identified SOZ and RZ in Figure 2A,B and the top 3 or 10 nodes as ranked by total GC outdegree in Figure 2F [1]. Figure 2E displays the largest magnitude GC map edge weights for reference. Figure 3 and Figure 4 are analogous to Figure 2 and display additional patients offering diversity of seizure types. Refinement of a method to select a SOZ from these ranks would represent the final step towards theoretical creation of a single-stage algorithmic basis for determination of SOZ in preparation for epilepsy surgery. Nevertheless, allowing for ease of comparison to the literature, this study displays results via the rank order sum method, leaving this last step for future study.

## 3. Results

**Figure 5:**
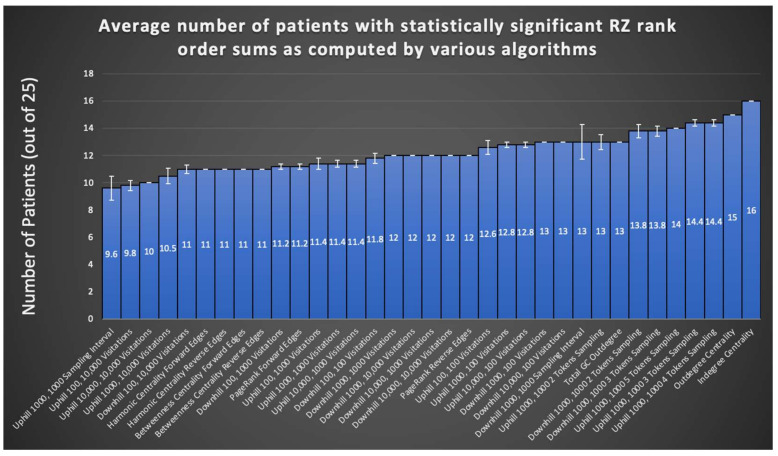
The RZ rank order sums of all algorithms and their variants are displayed here, with total GC outdegree included for comparison. SE bars are shown from sample sizes of five runs for each algorithm. Note the results of in/outdegree centrality algorithms, which exceed that of the total GC outdegree method from the literature while avoiding stochasticity of results found in other tested graph algorithms.

A summary evaluation of all graph algorithms tested via the rank order sum method for RZ is provided in Figure 5. Displayed are the number of patients, out of the 24 retrospectively tested, that exhibited a significant rank order sum for the RZ (one patient did not undergo resection surgery after completing invasive monitoring). Each algorithm is run at least five times on all patients in the sample and the average of all runs for an algorithm is shown. Certain runs of the Monte Carlo Sampling algorithm variant that keeps track of the visitation interval achieve statistically significant rank order sums for 16 out of 24 patients’ RZ, representing an improvement of three patients from methods in the literature [1]. As seen in Figure 5, in general, sampling algorithms utilizing more than one token achieved a larger number of patients with statistically significant rank order sums than sampling with a single token, suggesting that tracking the convergence of multiple tokens eliminates much of the noise found in random sampling. However, as can be seen from the SE depicted by error bars in Figure 5, there is significant variation between runs in most variants of the Monte Carlo Sampling algorithm, likely limiting its overall usefulness for surgery planning.

PageRank offers consistency of results from run to run, yet does not improve from the literature on the number of patients with a statistically significant rank order sum. Nevertheless, the fact that such a widespread algorithm used for other means in society can also be applied to the problem of seizure onset prediction opens the possibility for exploration of other graph algorithms originally invented for other purposes.

Combining the best of PageRank and Monte Carlo Sampling, indegree and outdegree centrality combined consistency of results with an improvement on the number of patients exhibiting statistically significant rank order sums. Notably, the 13 patients with statistically significant RZ rank order sums from Park and Madsen, 2018, do not completely overlap with the corresponding set of patients for graph algorithms tested in this study. Of the 16 significant patients from the most successful Monte Carlo sampling run, 10 patients are also part of the 13 from the total GC outdegree method from the literature. However, of the 16 significant patients from indegree centrality, 12 are also part of the 13 from total GC outdegree. Nevertheless, the lack of complete overlap suggests a combination of these methods could yield greater RZ and SOZ predication accuracy.

**Figure 6:**
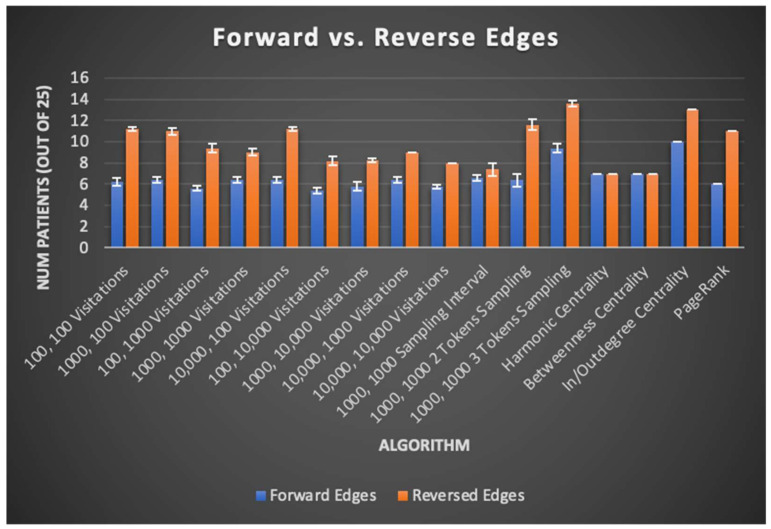
In this chart, sampling algorithms are described by the number of random restarts and the number of samples taken per random restart. Algorithms utilizing GC maps with forward edges include downhill sampling and indegree centrality. Algorithms utilizing GC maps with reverse edges include uphill sampling and outdegree centrality. Across nearly all algorithms tested, graph algorithms run on a reversed GC map had higher SOZ predictive capability, suggesting that interictal brain regions that cause neural activity in other regions better corelate with the SOZ rather than brain regions with the resulting neural activity itself.

There was minimal improvement in SOZ rank order sums (13.6 patients on average versus 13 for total GC outdegree), likely limiting the immediate clinical potential of any of the algorithms tested. Nevertheless, important findings are derived from comparison of the graph algorithms’ results across their many variants. One such finding is displayed in Figure 6 and discussed further below, where algorithm variants “reversing” edges to identify the trigger nodes of neural activity instead of the nodes exhibiting that activity itself almost universally perform better than their “forward” counterpart algorithms. For further details, results from each trial of centrality, PageRank, and total GC outdegree algorithms are reported in Table S3.

## 4. Discussion

This study is the first to the authors’ knowledge that applies graph algorithms to Granger Causality analyses to holistically consider baseline interictal EEG data for RZ/SOZ detection, using approaches which can be influenced by the topology of nodes’ connections and not just the sum of their GC outdegrees. By demonstrating the comparable utility of these algorithms to those from the literature, we hope to motivate future refinement of other algorithm variants that take this holistic lens. Especially when different graph algorithms yield statistically significant rank order sums for different subsets of patients, further exploration of these algorithms has the potential to exhibit predictive capabilities for patients not yet covered by any previous algorithms.

Other contributions of this study to algorithmic seizure onset and resection zone identification via EEG data are the patterns it reveals in seizure network topography across patients. Namely, the effects of edge direction on algorithm performance reveal that the brain region influential in triggering neural activity better correlates to the SOZ as opposed to the brain regions exhibiting triggered neural activity itself. This observation holds true for Monte Carlo sampling, centrality, and PageRank algorithms, as seen through their tested variants. Uphill sampling, outdegree centrality, and reversed edges PageRank, all of which can be said to have “reversed edges”, give a higher rank to the root cause of neural activity rather than the regions exhibiting activity itself. These patterns are summarized in Figure 6, where uphill/outdegree edge directions universally correspond to equal or better patient SOZ identification outcomes. This result is intuitive, given that the goal of surgery might be expected to be removal of high generators, rather than receivers, of causative signals. This result is also in line with previous studies based on the total GC outdegree method, which gives a higher rank to nodes that have greater total outward edge weights, likely indicating such nodes are more influential in the generation of activity in a seizure network [1].

Additionally, we find that stochasticity in many of the algorithm variants in this paper impacts patient results to the point where predictions about which nodes are to be included in the SOZ and RZ are no longer reliable. This point is proven by the success of sampling algorithms with multiple tokens and the requirement that numerous tokens have to simultaneously be present in order to record a visitation. Such an algorithm eliminates the effects of randomness and keys future algorithm variants to be developed to do the same.

Finally, all of the algorithms tested in this study reveal that seizure activity truly is localized to a specific brain region. The relative success of recording nodes’ length of intervals between sampling as opposed to nodes’ overall number of visitations shows this, as sampling intervals seek to tease out cyclical brain activity. This is corroborated by the success of indegree and outdegree centrality compared with betweenness and harmonic centrality. The latter two identify patterns in a node’s role in the context of the entire brain network, because they look at shortest paths between all pairs of nodes. On the other hand, indegree and outdegree zero in on the localized role of a node in relation to only its neighbors. The success of the indegree and outdegree approaches exemplifies the need to focus on localized areas of the brain network instead of approaching all nodes together.

All of these insights were achievable only with information told by the edge weights contained in GC maps, data which were previously discarded. As data becomes available, future work will involve a greater number of patients than the 25 included in this study, in order to better determine the applicability of GC to a wider patient population. These additional patients will include those with sEEG implants as well as more ECoG cases similar to the 25 already studied. Future directions of this work include identifying the interictal time window when GC map-based SOZ and RZ predictions are most accurate. Because we obtain 60 different GC maps selected at random for each patient, these maps can be tagged with time elapsed since previous ictal activity and time to next ictal activity. With this data, an optimal window during which predictions most resemble the actual SOZ and RZ can be identified.

## 5. Conclusions

In this retrospective study, we develop and test new algorithms based on the entire network as revealed by GC, rather than simply each node’s outdegree. Within our cohort using these approaches, we find informative trends. Firstly, the matching of and improvement on algorithms in the literature by this study’s graph algorithms helps to solidify the relevance of GC maps to epilepsy surgery planning. Secondly, uphill sampling, outdegree centrality, and reversed edges PageRank, all of which can be said to have “reversed edges”, give a higher rank to the root cause of neural activity rather than the regions exhibiting activity itself. Thirdly, stochasticity in some algorithms impacts results such that predictions about nodes to include in the SOZ/RZ are no longer reliable. As such, variants that eliminate stochasticity such as sampling with multiple tokens greatly increase the usefulness of these highly random algorithms. Fourthly, the success of recording nodes’ sampling intervals as opposed to nodes’ overall number of visitations, as well as the success of indegree and outdegree over betweenness and harmonic centrality, reveal that seizure activity tends to localize to a specific brain region. Future work will involve more patients, including sEEG patients, to fine-tune the applicability of GC-based graph algorithms to a wider patient population.

## Data Availability

The patient datasets analyzed for this study can be provided upon request. The data are not publicly available due to patient data compliance restrictions. Sample code used to execute graph algorithms and the Monte Carlo rank order sum simulations can be found at https://osf.io/z3y9e/?view_only=f891e593a14a4f468819a491f829e9d6; (accessed on 17 June 2021).

## Supplementary Materials

The following are available online at https://www.mdpi.com/article/10.3390/brainsci11091167/s1, Table S1: Demographic information and clinical outcomes for 25 patients included in this study’s dataset; Table S2: Additional clinical and seizure localization data for 25 patients included in this study’s dataset; Table S3: Results from each trial of the centrality, PageRank, and total GC outdegree algorithms attempted. Note that there are 25 total patients for the SOZ results and 24 total patients for the others as only 1 patient did not undergo resection surgery after long-term monitoring.
