# Supplementary material for: Extensions of Granger Causality Calculations on Brain Networks for Efficient and Accurate Seizure Focus Identification via iEEGs
Source: Brain Sci. 2021 Sep 1;11(9):1167. doi: 10.3390/brainsci11091167 (PMC8471554; doi:10.3390/brainsci11091167)
Supplement: Supplementary file 1 [file brainsci-11-01167-s001.zip › brainsci-1284338-supplementary.pdf]

Supplementary Material

Supplementary Figures and Tables

Table S1. Demographic information and clinical outcomes for 25 patients included in this study's dataset

Patient No.	Age/gender	Etiology	Pathology	Resection procedure	Engel class
1	8/F	Cortical dysplasia of the right inferior frontal gyrus	Gliosis and dysplastic neurons	Right frontal lesionectomy	Ia
2	2.25/M	Left frontal lesion	FCD Type IIB, Low grade glial neoplasm	Resection of tumor and small additional resection of cortex adjacent to the cavity	Ia
3	18/F	Unknown	Gliosis	Left mesial temporal nonlesional resection	Ia
4	18/M	Unknown	Gliosis	Left anterior and mesial temporal resection	Ia
5	18/F	Extensive bilateral heterotopia	FCD Type IA, Hippocampal gliosis	Right temporal resection	Ia
6	19/F	Lesion in left superior temporal gyrus	WHO Grade I-II Ganglioglioma	Resection of tumor in the superior temporal sulcus	Ia
7	11/M	Unknown	FCD Type IIIA, Gliosis	Left anterior temporal resection and additional resection of lateral and basal seizure focus	III
8	10/F	Right medial frontal DNET	Recurrent/residual low-grade glioma	Resection of parasagittal brain tumor	Ia
9	10/M	Focal cortical dysplasia; Prior year left frontal resection	FCD Type IIA, Gliosis	Extension of prior resection, four years earlier, of a left frontal cortical dysplasia	Ia
10	16/M	Unknown	MTS, Gliosis	Left anterior mesiotemporal resection	Ia
11	2/F	Unknown	Microglia activation, Gliosis, Neuronal injury	Left parietal and frontal cortex resection	Ia
12	6/M	Left frontal focal cortical dysplasia	FCD Type IIA	Functional disconnection of left frontal lobe anterior to motor strip	Ia

13	20/F	Right parietal oligodendroglioma	Cerebral gray and white matter with extensive reactive changes (oligodendroglioma 11 years earlier, then gliosis 4 years earlier)	No resection was done due to concern over motor deficit	N/A
14	10/M	Unknown (focal seizures, epileptic encephalopathy)	Cortex and white matter with reactive changes	Right frontal resection	Ia
15	16/F	Left temporo-occipital subcortical heterotopia	FCD Type IIA, Vascular malformation	Resection of seizure focus on the left side	Ia
16	17/M	Left mesial temporal lesion	FCD Type IIA, HS	Left temporal lobe and medial structures resection	Ib
17	12/M	Left mesial temporal sclerosis, Left temporal dysplasia	FCD Type IIA, HS, Gliosis	Left mesial temporal lobe resection	Ia
18	13/F	Unclear: MRI scans suggest some possible cortical dysplasia in the anterior-superior temporal gyrus	FCD Type IIA, Gliosis	Partial left temporal lobectomy	Ib
19	18/M	Presumed cortical dysplasia of left para-hippocampal gyrus (MTLE)	MTS, Gliosis	Left temporal tip and mesial temporal resection	Ia
20	10/M	Right MCA in utero stroke affecting inferior frontal temporal and parietal areas with intraparenchymal cyst and encephalomalacia	FCD Type IA, Gliosis	Extension of resection of right frontal cortical dysplasia (prior surgery 5 years earlier)	Ia
21	10/F	Right Frontal lobe lesion (medial superior)	FCD Type IIB	Resection of right mesial frontal lesion in the vicinity of the motor strip	Ia

22	18/M	Nonlesional, left frontal	Irregularities of cortical development	Left mesial frontal resection	Ia
23	8.5/F	Suspected right parasagittal cortical dysplasia	Irregularities of cortical development, Gliosis	Resection of large cortical dysplasia, duraplasty	Ia
24	2/M	TSC due to a de novo TSC2 mutation	Severe dysplasia and abnormal glioneuronal cells	Right frontal resection for multiple subcortical tubers	Ia
25	18/F	Presumed left frontal cortical dysplasia	FCD Type IIB	Left frontal cortical resection	II

Table S2. Additional clinical and seizure localization data for 25 patients included in this study's dataset.

Patient No.	Seizure Types	Duration	Frequency	Longest Seizure-Free Interval	Semiology	MRI	EEG
1	Clonic arm extension, behavioral arrest, repetitive speech	20-40 secs	5-10/day	2 yrs	Frontal vs. temporal lobe	R frontal inferior gyrus	Regional, R fronto-temporal onset
2	Right arm clonic jerking, clonic facial jerks	5-10 secs in clusters	6-10/day (max)	None	L frontal	L frontocentral	L frontocentral
3	Altered consciousness, purposeless movements, aphasia, lip smacking, confusion	1-2 mins			Non-lesional L temporal		
4	Generalized convulsive, complex partial (staring, mumbling, sucking teeth)		3-5/month	18 months		Lesion adjacent to L temporal tip	
5	Deja vu phenomenon, urinary incontinence; oral and hand automatisms, stomachache		5/day (max)	Few weeks		Mostly occipital and temporal	
6	Generalized convulsive with urinary incontinence; sound distortion, fear, nausea	20-60 secs	1/day; 4-5/day max	2 weeks			
7							
8	GTC, left arm extension, head version left, entire left side stiffening		Daily (max)	9 months		Lesion R frontal lobe	
9	Whole body stiffening, with tremor/clonic	Cluster of ~50 lasting 20 secs - 2		2 yrs			

	movements of the upper and lower extremities	mins; prolonged 1 hour					
10	Staring, sitting up, perioral cyanosis, stiffening, sucking of lips		7 in 10 hrs (max)	1 month	L temporal	L anterior temporal	L anterior temporal
11	Tongue twitching, R mouth deviation, flexion of arms, clenching of fists, urinary incontinence	30 secs - 10 mins			L hemisphere (L perisylvian)	Non-localizing	L frontal
12	Clusters of twitching of the R eyelid, hand, and leg	up to 1 min	3-4 clusters/day of 7-8 seizures each (max)	1.75 yrs	Gen & L sided	L frontal	L sided predominance
13	Simple partial seizure (L hand floppy, drops objects); simple partial -> complex partial seizure	5-10 secs (simple partial)	2+/week simple partial type; 0-3/month complex partial type	2 yrs	R hemisphere	R parietal lesion	R frontal
14	GTC seizures, L hand, face, eye, mouth	1-2 mins			R parasagittal, likely frontal region	Non-lesional	R-sided
15	Staring, decreased responsiveness, repetitive phrases	1-2 mins	1-2/wk	5 months	Temporal	L temporal	L temporal
16	Facial and upper body flushing, dysphagia, anxiety		10-15/day (max)	2-3 weeks	L temporal		
17	Stomach, stopping of movements; Neck stiffening, head turn, bilateral upper extremity stiffening and flexion	15-30 secs	0-5/day	Several months	L temporal	L mesial temporal sclerosis, dysplasia L temporal lobe	L anterior temp at LTM, L frontal / midline on ambulatory EEG
18	Déjà vu, staring, pausing, hand movements, incomprehensible speech; aphasic seizures difficulty breathing, sialorrhea, difficulty speaking	few mins to a few hours	4-5/week across all types	9 months	L temporal	L temporal	L temporal
19	Blank stare, fidgeting	30 secs - 2 mins	2-3/day	5 weeks	Temporal, non-lateralizing	L ant temporal / parahippocampal	L temporal (mid and anterior)
20	GTC seizures; staring; upward eye deviation, body trembling, lip smacking	few secs to couple mins	2-5 shorter ones / day; <3 longer ones / wk	3 yrs	R or non-localizing	R	R frontal > bilateral

21	L arm tonic flexion with or without aura; sometimes spreads to other parts	5-20 secs	5-6/night (max)	2 weeks	Frontal	R frontal lesion	Poorly localizing, possibly frontal
22	Twitching of R shoulder, R arm jerks, biceps twitch, gasping	1-6 secs	Daily (max)	hours	L frontal	Non-lesional	L frontocentral
23	Head version and eye deviation to L, L arm and leg stiffening, unresponsiveness; L sided Todd's paralysis		~18/month		R	R parietal	R centroparietal
24	L arm extension, R arm flexed at elbow, hunched forward, L eye flutter; tonic component with abdominal flexion, head nod, heavy breathing	10-120 secs	12/day (max)	1 week	R mesial fronto-central or R lateral frontal	Multifocal, large tuber R frontal lobe	R fronto-central
25	Gagging, coughing, facial twitching, unable to speak	20 secs - 2 mins	1-2 week		Non-localizing, L perisylvian fissure	L frontal cortical dysplasia; nonspecific R parietal signal abnormality	Non-localizing

Table S3. Results from each trial of the centrality, PageRank, and total GC outdegree algorithms attempted. Note that there are 25 total patients for the SOZ results and 24 total patients for the others as only 1 patient did not undergo resection surgery after long-term monitoring.

Direction	Algorithm	Significant Patients (SOZ)	Significant Patients (RZ)	Significant Patients (SOZ ∩ RZ)
Forward Edges	Harmonic Centrality	7	11	5
Reverse Edges	Harmonic Centrality	7	11	5
Forward Edges	PageRank	6	12	9
Reverse Edges	PageRank	11	12	6
Forward Edges	Betweenness Centrality	7	11	5
Reverse Edges	Betweenness Centrality	7	11	5
Forward Edges	Indegree Centrality	10	16	10
Forward Edges	Outdegree Centrality	13	15	10
N/A	Total GC Outdegree	13	13	9
